# One stage resection of spontaneous rupture of hepatocellular carcinoma in the triangular ligament with diaphragm invasion: case report and review of the literature

**DOI:** 10.1186/1749-7922-7-30

**Published:** 2012-09-21

**Authors:** Kwang-Kuk Park, Song-I Yang, Myung-hee Yoon

**Affiliations:** 1 Department of Surgery, Kosin University College of Medicine, Busan, South Korea

**Keywords:** Spontaneous rupture, Hepatocellular carcinoma, Hemoperitoneum

## Abstract

A spontaneous rupture of hepatocellular carcinoma (HCC) can lead to extensive hemorrhage and is a rare but life-threatening event. A 58-year-old male patient with no history of trauma presented at our institution with severe epigastric pain and abdominal distension for 6 h. His blood pressure was a 60/40 mmHg, and pulse rate was 132/min. Abdominal contrast enhanced computed tomography (CT) imaging revealed a ruptured mass under the left diaphragm and fluid collection in the upper abdomen, flanks and pelvic cavity. Exploratory laparotomy confirmed the presence of an active bleeding tumor in the triangular ligament invading into the diaphragm. The tumor was resected with an appropriate diaphragm margin. The resected tumor was 5 cm in diameter and pathologically identified as hepatocellular carcinoma with a negative surgical margin. This case report shows that ruptured hepatocellular carcinoma should be considered in the differential diagnosis of non-traumatic hemoperitoneum. And it is necessary to set a surgical plan for unpredictable HCC rupture with direct diaphragm invasion.

## Introduction

Hepatocellular carcinoma (HCC) is the fifth most common type of cancer diagnosed worldwide and the third leading cause of cancer-related mortality [1,2]. Spontaneous rupture is reported to occur in 3 – 15% of cases and is one of the most severe complications of HCC [3-5]. The prognosis for spontaneous rupture of HCC is poor, with a hospital mortality rate ranging from 33 to 67% [6-8]. However, clinical diagnosis of this HCC complication is difficult due to its atypical symptoms. For example, some patients may present with abdominal pain, abdominal distension and anemia, while others suffer from shock as the initial symptom. Furthermore, treatment of HCC is complicated by co-morbidities, coagulopathy, hemorrhagic shock, liver cirrhosis, and decreased liver function. A peripherally located large HCC lesion is clinically prone to grossly involve the diaphragm, either by dense adhesion or as a result of histological invasion [9]. According to autopsy studies, direct diaphragmatic involvement is found in 10%–13% of patients with HCC [10], and in such cases, *en bloc* resection of the diaphragm seems appropriate. However, such extensive surgery was thought to present too high a risk of damage during the postoperative course. This case study looks at a previously undiagnosed patient with a spontaneously ruptured HCC in triangular ligament with diaphragm invasion.

## Case report

**Figure 1:**
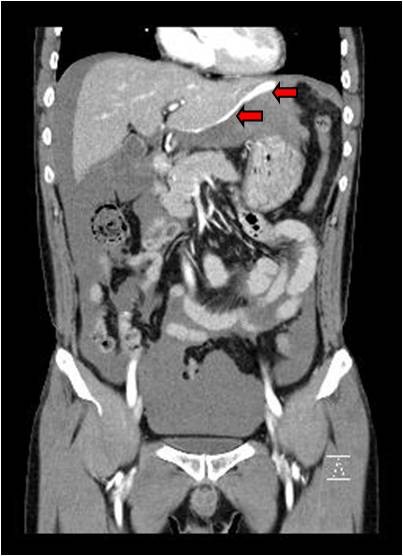
A contrast extravasation is shown from a mass like lesion on the lateral border of the liver (arrow) and hemoperitoneum.

**Figure 2:**
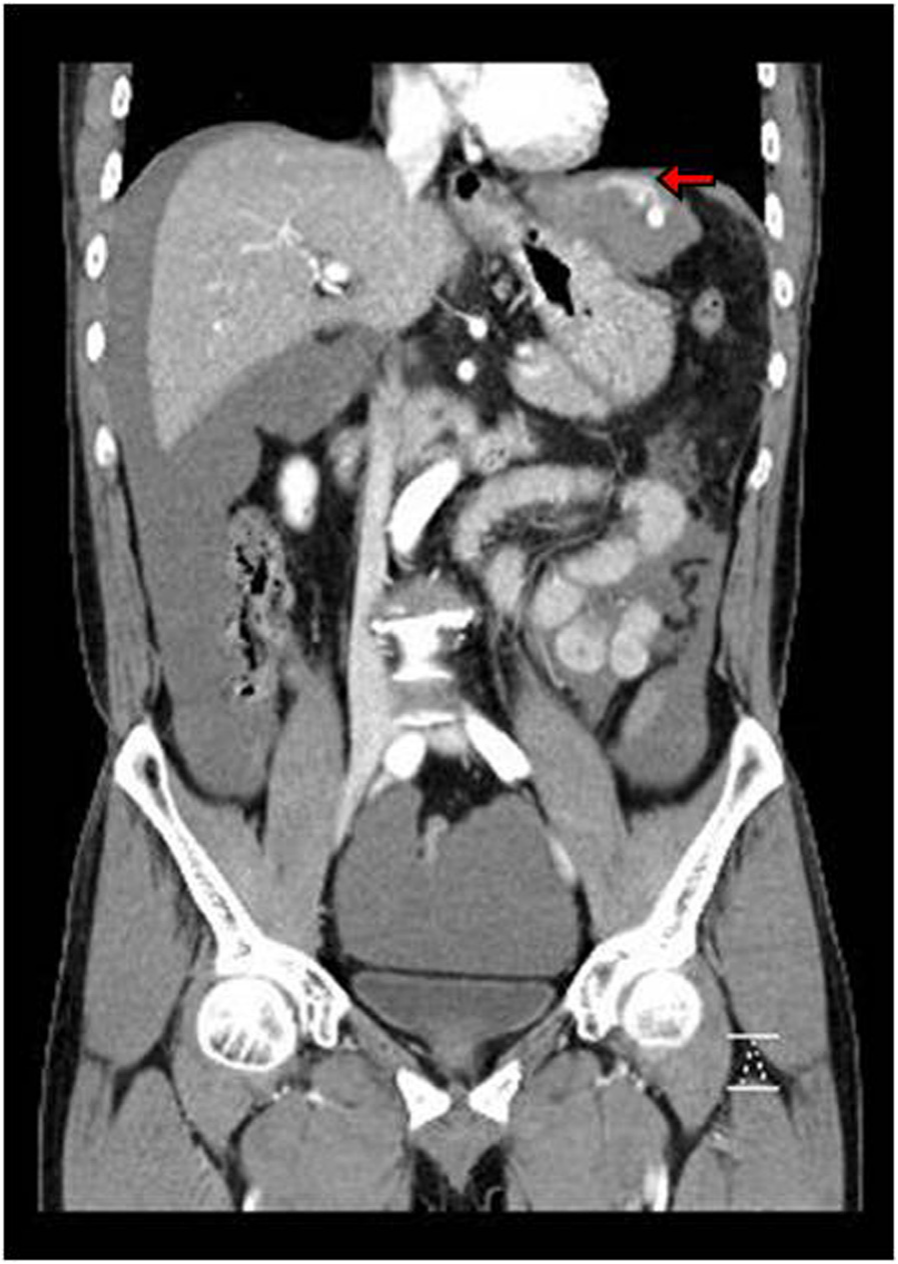
Abdominal CT showes diaphragm invasion of the mass like lesion (arrow).

**Figure 3:**
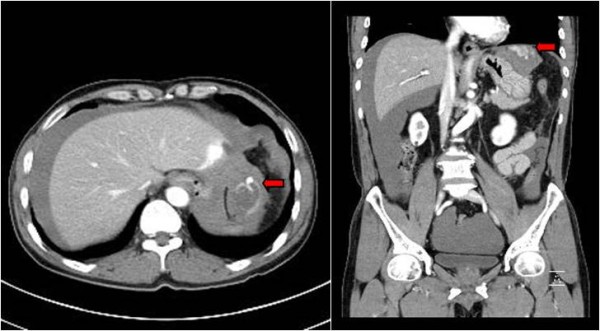
**A liver surrounded by a large volume of fluid is seen.** An approximately 4cm sized low density lesion is located in the periphery of the lateral segment (arrow).

A 58-year-old man visited the emergency department with hypovolemic shock. His chief complaint was the sudden onset of epigastric pain with abdominal distension lasting 6 h. Physical examination revealed an ill appearance with a blood pressure of 60/40 mmHg and a pulse rate of 132/min. Conjunctiva were pale but not icteric. Breath sounds were clear, and heart sounds were regular and without murmurs. The patient had negative history of hepatitis B, hepatitis C or trauma. Hemoglobin was 6.9 g/dl, white blood count was 15,800/mm3 and platelet count was 176,000/mm^3^. Liver function tests were within the normal range [serum alanine transaminase 35 IU/l (normal: 5–40 IU/l), serum aspartate transaminase 18 IU/l (normal: 8–40 IU/l), gamma glutamyltranspeptidase 16 IU/l (normal:<30 IU/l), alkaline phosphatase 38 IU/l, total billibubin 0.6 mg/dl, direct billibubin 0.3 mg/dl]. Prothrombin time and International Normalized Ratio (INR) were prolonged with prothrombin time of 16.4 s (normal: 10.2 – 13.6), and INR of 1.43 (PT ratio). Abdominal contrast enhanced CT imaging revealed a mass invading the diaphragm with contrast extravasation in the left, lateral segment of the liver (Figure 1, and Figure 2). Fluid collection was found in the upper abdomen, subhepatic area, flanks, and the pelvic cavity. Imaging also revealed the presence of a ruptured abdominal mass (Figure 3). The exploratory laparotomy discovered 3000 ml of blood in the abdominal cavity. The liver was non-cirrhotic, and there was an actively bleeding invasive tumor in the left lateral triangular ligament of the liver. The tumor was resected with an appropriate margin and the specimen was histologically confirmed as a 5-cm HCC with negative margin. The post-operative course was unremarkable, and the patient was discharged on the 10th day post-surgery.

## Discussion

HCC is the most common primary malignant tumor of the liver [1,2]. Lai and W. Y. Lau analyzed literature published between 1970 and 2004 and found 1500 published cases of spontaneous HCC rupture [2]. This complication is observed in 3% of the Western population and in 14% of the Asian population, and mortality ranges between 25 and 75% [2,11]. The mechanism behind the spontaneous rupture of HCC remains unclear but a number of hypotheses to explain this phenomenon have been published. Possible etiological factors include subcapsular location, tumor dimensions, portal hypertension, tumor necrosis, local increase in venous pressure due to outflow reduction caused by neoplastic invasion, and previous vascular injury which might predispose a patient to HCC rupture and to the rupture of smaller lesions in other locations [12,13]. Usually, the initial symptom is sudden epigastric or right hypochondrial pain. Some patients present with shock, and most have signs of peritonitis, abdominal distension or both. Patients also often present paracentesis-positive with blood-stained ascites [14]. In the presented case, the patient complained of acute abdominal pain and distension. Preoperative diagnosis of HCC rupture is difficult in patients with no previous history of cirrhosis or HCC. Vergara *et al.* reported that an accurate preoperative diagnosis of ruptured HCC was predicted in only 25% of cases, despite shock being present in 33 – 90% of the patients [15]. Doppler ultrasound and CT imaging are useful in delineating hemoperitoneum and liver tumors and CT is specifically useful in detecting HCC rupture by visualizing the tumor and blood loss. The peripheral location and protrusion of the tumor and discontinuity of the hepatic surface and surrounding hematoma with high attenuation on CT are very helpful signs in the diagnosis of ruptured HCC [16]. In this case study, contrast extravasation of the tumor and pooling of blood at the periphery of the liver due to hemoperitoneum were helpful in establishing the diagnosis. The management of ruptured HCC is achieved by many techniques depending on the stability of the patient. If the patient is hemodynamically stable, conservative treatment with close monitoring and correction of coagulopathy is the gold standard of care [17]. On the other hand, if the patient is hemodynamically unstable, as in our case, he or she may need surgical interventions after resuscitation. These include transarterial embolization, perihepatic packing, suture plication, absolute alcohol injection, hepatic artery ligation (HAL) or emergency lobe resection. Surgical interventions also depend on the condition of the liver, the size of the tumor and its location. Perihepatic packing is preferred in a bleeding tumor located near the diaphragm but the packing should not be left in for more than 36–72 h due to risk of infection [2]. Tumor blood supply comes mainly from the hepatic artery, and the efficacy of HAL is estimated to be 68-100%, with mortality as high as 77% [2]. Due to the risk of liver damage, selective HAL is preferred. One-stage emergency liver resection simultaneously stops bleeding and definitively treats HCC. The resection index in patients with a ruptured HCC ranges between 12.5 and 31%, and these procedures have a high mortality rate due to inadequate knowledge of the functional hepatic reserve (hemorrhagic shock condition). Reported mortality ranges between 16.5 and 100%, depending on the institution [2] and many authors consequently prefer staged liver resection after initial bleeding control. The resection index mentioned above ranged between 21 and 56%, while postoperative morality was reported between 0 and 9%. Therefore, one-staged liver resection in ruptured HCC cases should only be performed in easily accessible tumors and only in patients without liver cirrhosis [2]. In our case, the diagnosis of HCC was accidental, and the patient had no history of hepatic disease. On admission, the patient was hemodynamically unstable but had normal liver function. Hemoperitoneum secondary to hepatic rupture was confirmed by CT imaging, and we proceeded with emergency surgery. However, the tumor’s advanced stage made it difficult to access and isolate since it was already infiltrating the diaphragm. Direct diaphragmatic invasion of HCC is found in 10% to 13% of patients with HCC [10]. To date, 7 retrospective studies and 2 case reports in the English literature report that a total of 162 patients with HCC direct invasion to the diaphragm have undergone *en bloc* resection or blunt dissection (Table 1). Lau *et al.* and Lin *et al.* reported no significant differences in the surgical morbidity and mortality between patients who underwent a traditional hepatectomy and those who had diaphragm resection [18,19]. Yamashita *et al*. reported no significant difference in short- or long- term surgical impacts between patients who received *en bloc* resection and those who had blunt dissection and suggested that surgeons should consider the possibility of tearing the HCC during blunt dissection between the tumor and the diaphragm [20]. Leung *et al.* have reported that a resection margin of 1 cm was the only significant prognostic factor for poor disease-free survival after *en bloc* resection [21]. However, Lin *et al.* pointed out that there was a possibility of increased intraoperative blood loss and a longer surgery when the diaphragm was resected [18]. Thus, it is necessary to set a surgical plan for unpredictable HCC rupture with direct diaphragm invasion in a situation of emergency laparotomy such as our case. In our case, the patient was saved by the prompt identification of the ruptured HCC and good liver function without liver cirrhosis.

**Table 1:** Reports on diaphragm invasion of HCC

**Author**	**Year**	**Number of cases**	**En bloc resection or Blunt dissection**
Jeng et al. [22]	1994	8	En bloc resection (all)
Wu et al. [23]	1994	14	N/A^1^- Preoperative TAE and resection (all)
Lau et al. [19]	1995	14	En bloc resection (all)
Tung et al. [24]	1996	16	En bloc resection (all)
Leung et al. [21]	2001	28	En bloc resection (all)
Lin et al. [18]	2005	53	En bloc resection (all)
Kaur et al. [25]	2008	1	En bloc resection
Yamashita et al. [20]	2011	27	En bloc resection (n =13) Blunt dissection (n = 14)
Maruyama et al. [26]	2012	1	En bloc resection

^1^not available.

## Conclusion

The prognosis of spontaneous rupture of HCC is poor with a high hospital mortality rate. A peripherally located large HCC lesion is clinically prone to grossly involve the diaphragm, either by dense adhesion or as a rare result of histological invasion. In such cases, *en bloc* resection of the diaphragm seems appropriate; however, such extensive surgery is thought to present too high a risk of damage during the postoperative course, especially in emergency operation. For hemoperitoneum patients with unpredictable HCC rupture and diaphragm invasion, physicians should establish a therapeutic plan with consideration of a surgical approach.

## Consent

Written informed consent was obtained from the patient for publication of this case report and any accompanying images. A copy of the written consent is available for review by the editor-in-chief of this journal.

## Competing interests

The authors declare that they have no competing interests.

## Authors’ contributions

MHY coordinated the team, helped in literature research and edited the final version of the manuscript. PKK collected the information and wrote the article SIY researched the literature and wrote the article. All authors read and approved the final manuscript.
